# Supplementary material for: Marine introgressions and Andean uplift have driven diversification in neotropical Monkey tree frogs (Anura, Phyllomedusinae)
Source: PeerJ. 2024 Apr 16;12:e17232. doi: 10.7717/peerj.17232 (PMC11027904; doi:10.7717/peerj.17232)
Supplement: Supplemental Information 4 — This includes explanations on how we attributed the values in the matrix and rules of “dispersal penalties” based on the landscape evolution in an attempt to minimize the impact of the arbitrary choice. Also includes the table of penalties and the time-stratified dispersal matrix. Table S4.1 – Penalties in the construction of the time-stratified dispersal matrices (TS). Numbers indicate the penalty in the dispersal probability in the time-stratified matrix (TS; see Table S3.2 below). R are the penalties for reaching the barrier and C are for crossing. Red cells indicate maximum penalty, meaning that is impossible to reach/cross that barrier. Table S4.2 – The time-stratified dispersal matrix (TS). TS is separated by timeframes. Letters in the columns and lines represents the units utilized in the work (see Fig. 1 for more details). Letters in the first column represent the units from where the dispersal came, and letters in the first line represent the dispersal destination. [file peerj-12-17232-s004.docx]

*PeerJ*

**SUPPLEMENTARY MATERIAL**

**Marine introgressions and Andean uplift drives diversification in neotropical Monkey tree frogs (Anura, Phyllomedusinae)**

Diego Almeida-Silva, Leonardo Matheus Servino, Matheus Pontes-Nogueira, Ricardo J. Sawaya

Correspondence: leonardo.servino@ib.usp.br

**Appendix S4 – Explanation on how the time-stratified dispersal matrix was built. This includes explanations on how we attributed the values in the matrix and rules of “dispersal penalties” based on the landscape evolution to minimize the impact of the arbitrary choice. Also includes the table of penalties and the time-stratified dispersal matrix.**

**PENALTIES FOR DISPERSAL**

The use of time-stratified matrices on BioGeoBEARS allows the inclusion of landscape evolution information, improving biogeographical models concerning the level of detail (Matzke, 2013, 2014). Values written on each matrix vary from 0 to 1, to multiply the dispersal probabilities (dp) between two areas for a given time slice. Once they are chosen arbitrary, we exploit this functionality to establish some criteria of penalties among areas, accounting distance, geographical barriers, and the non-stationarity of landmasses acting on species dispersal, to minimize the effects of this arbitrariness.

In the time-stratified matrix (TS), we considered that, if there was no barrier, the dispersal probability between any two areas would be 1, as it would indicate to the model that dispersal between two areas would not be interfered. However, barriers do exist, so this initial value of 1 exists only between areas that do not have any barrier considered in the work. If a dispersal route passes through a barrier, we discount the value in the Table S3.1 from this initial value of 1, resulting in the values presented at TS (Table S3.2). For example: the Eastern and Central Cordilleras of Andes influences the dispersal between cis and trans-Andean regions. We considered that dispersal probability between 0 and 3 millions of years would have a penalty of 0.5 between these regions, as a specie would have to cross the Cordillera. So, if a dispersal occurs between these regions, dispersal probability at TS in this timeframe would be 1 minus 0.5 (1 – 0.5 = 0.5). The values can stack up, as some dispersal routs can pass through multiple barriers (e.g., Andean Cordillera and Amazon River, that would be 1 – 0.5 – 0.1 = 0.4). We summarise this information in Table S3.1, detailing below:

**Criteria of penalties in Neotropical Region**

We applied a maximum penalty in cases where landmasses were isolated by the ocean, preventing dispersal among areas (i.e., setting dp = 0.0). This scenario was observed previously to the formation of Isthmus of Panama during 55–23 millions of years ago (mya), and so we consider South and Central America were not connected in respective timeslices. We reduced penalties thereafter in subsequent timeslices, increasing the dp as the two continents became closer and started to collide approximately 15 mya (Hoorn *et al.*, 2010; Farris *et al.*, 2011; Bacon *et al.*, 2013, 2015; Jaramillo *et al.*, 2017).

The occurrence of marine introgressions was quite common in the evolution of south-american landscape over the past 50 mya. Some of the most well-documented of these events occurred during Miocene, as the Paranean Sea, Pebas and Acre Systems (Hernández *et al.*, 2005; Hoorn *et al.*, 2010; Salas-Gismondi *et al.*, 2015; Boschman & Condamine, 2022). To keep our biogeographical hypothesis simpler, and considering these marine introgressions flooded large landmasses, we established different penalties for a species to cross or inhabit these areas. By default, we set a penalty of 0.5 to cross marine introgressions, and half of this value (0.25) to inhabit. Again, we reduce penalties thereafter in subsequent timeslices when it was appropriate.

On the other hand, the uplift of mountain ranges represents a progressively increasing geographical barrier over time. We deal with this scenario twice in our analyses. Firstly, regarding the uplift of Eastern and Central Cordilleras of Andes along Miocene (Hoorn, 1993; Gregory-Wodzicki, 2000). Since this mountain range acts as climatic and physical barrier to Phyllomedusinae, we multiply dp by progressively smaller values, increasing the penalty through time. However, we set a lower penalty (0.25) to the late events acting on the uplift of Serras do Mar and Mantiqueira Mountain Ranges (e.g., Souza *et al.*, 2021). In detail, penalties were applied only to species crossing both geographical barriers, once they represent a huge geological formation in southeastern Brazil. Due to the incredible diversity of phyllomedusids occurring in Serra do Mar region, in association to climatic conditions present in tropical rainforest, we did not apply penalties to species inhabiting.

The major rivers in Amazonia can also act as geographical barriers to dispersal. Hence, we applied penalties to dp in species whose distributions are related to these rivers. Consequently, species had a cost of 0.1 to cross Amazonas (dispersal C ⇆ D and D ⇆ E) and Madeira (dispersal C ⇆ E) rivers.

Finally, the emergence of South American dry diagonal during the late Pleistocene and Holocene ends a connection between Amazonian rainforest and Atlantic rainforest. Due to its landscape, we set penalties to species cross and inhabit this area. Moreover, we increased these values through time, with the consolidation of the South American diagonal of open/dry landscapes.

**Criteria of penalties in Oceania**

Dispersals between Neotropical and Australasian landmasses was enabled by the connection between the two regions and Antarctica, both completely lost between late Eocene and early Oligocene (35–30.5 Mya). For being a process through time instead a one-off event, we successively increase the penalisation along timeslices until the maximum value. We also applied the maximum penalty in the case of landmasses that has experienced complete inundation through time.

**Table S4.1 – Penalties in the construction of the time-stratified dispersal matrices (TS).** Numbers indicate the penalty in the dispersal probability in the time-stratified matrix (TS; see Table S3.2 below). R are the penalties for reaching the barrier and C are for crossing it. Barriers and numbers in bold indicate where there is maximum penalty, meaning that is impossible to reach/cross that barrier.

| Barriers | Timeslices (Millions of years) | | | | | | | | | | | | | |
| --- | --- | --- | --- | --- | --- | --- | --- | --- | --- | --- | --- | --- | --- | --- |
|  | 0-3 | | 3-7 | | 7-10 | | 10-23 | | 23-35 | | 35-45 | | 45 - 55 | |
|  | R | C | R | C | R | C | R | C | R | C | R | C | R | C |
| (a) Neotropical Region | | | | | | | | | | | | | | |
| Amazonas/Madeira rivers | 0 | 0.1 | 0 | 0.1 | 0 | 0 | 0 | 0 | 0 | 0 | 0 | 0 | 0 | 0 |
| **Isthmus of Panama** | 0 | 0 | 0 | 0.25 | 0 | 0.5 | 0 | 0.75 | **1** | **1** | **1** | **1** | **1** | **1** |
| Northern Andes | 0.25 | 0.5 | 0.25 | 0.5 | 0.15 | 0.25 | 0.05 | 0.15 | 0 | 0 | 0 | 0 | 0 | 0 |
| South American Dry Diagonal | 0.25 | 0.5 | 0.15 | 0.25 | 0 | 0 | 0 | 0 | 0 | 0 | 0 | 0 | 0 | 0 |
| Serras do Mar/Mantiqueira Mountain Range | 0 | 0.25 | 0 | 0.25 | 0 | 0.25 | 0 | 0 | 0 | 0 | 0 | 0 | 0 | 0 |
| Acre System | 0 | 0 | 0 | 0 | 0.25 | 0.5* | 0 | 0 | 0 | 0 | 0 | 0 | 0 | 0 |
| Pebas System | 0 | 0 | 0 | 0 | 0 | 0 | 0.25 | 0.5 | 0 | 0 | 0 | 0 | 0 | 0 |
| Paranaense Sea | 0 | 0 | 0 | 0 | 0 | 0 | 0.25 | 0.5 | 0 | 0 | 0 | 0 | 0 | 0 |
| (b) Oceania | | | | | | | | | | | | | | |
| **Loss of connection with Antarctica** | **1**** | **1**** | **1**** | **1**** | **1**** | **1**** | **1**** | **1**** | **1**** | **1**** | 0 | 0.5 | 0 | 0.25 |

*Crossing from D to E and F suffered an average discount (0.25) because the Acre system divides the D region so that the connection between the areas is divergent between north and south.

**Dispersion between Australia and South America becomes impossible after the loss of connection between the two continents with Antarctica.

**Table S4.2 – The time-stratified dispersal matrix (TS).** TS is separated by timeframes. Letters in the columns and lines represents the units utilized in the work (see Fig. 1 for more details). Letters in the first column represent the units from where the dispersal came, and letters in the first line represent the dispersal destination.

| 0 - 3 milion years ago | | | | | | | | | | | | | |
| --- | --- | --- | --- | --- | --- | --- | --- | --- | --- | --- | --- | --- | --- |
|  | | Destination | | | | | | | | | | | |
|  |  | A | B | C | D | E | F | G | H | I | J | K | L |
| From where they came | A | - | 0.75 | 0.4 | 0.4 | 0.2 | 0.1 | 0.1 | 0.1 | 0.1 | 0.1 | 0.3 | 0 |
|  | B | 0.75 | - | 1 | 1 | 0.8 | 0.7 | 0.45 | 0.45 | 0.7 | 0.8 | 0.9 | 0 |
|  | C | 0.4 | 0.75 | - | 0.9 | 0.9 | 0.8 | 0.55 | 0.55 | 0.8 | 0.9 | 1 | 0 |
|  | D | 0.4 | 0.75 | 0.9 | - | 0.9 | 0.8 | 0.65 | 0.4 | 0.6 | 0.7 | 0.8 | 0 |
|  | E | 0.2 | 0.55 | 0.9 | 0.9 | - | 1 | 0.75 | 0.65 | 0.8 | 0.9 | 1 | 0 |
|  | F | 0.1 | 0.45 | 0.8 | 0.8 | 1 | - | 0.75 | 1 | 0.9 | 0.75 | 0.65 | 0 |
|  | G | 0.1 | 0.45 | 0.8 | 0.8 | 1 | 1 | - | 1 | 1 | 1 | 1 | 0 |
|  | H | 0.1 | 0.1 | 0.2 | 0.2 | 0.4 | 0.75 | 0.75 | - | 1 | 0.75 | 0.65 | 0 |
|  | I | 0.1 | 0.1 | 0.2 | 0.2 | 0.4 | 0.75 | 0.75 | 1 | - | 1 | 0.9 | 0 |
|  | J | 0.1 | 0.1 | 0.2 | 0.2 | 0.4 | 0.75 | 0.75 | 0.75 | 1 | - | 1 | 0 |
|  | K | 0.3 | 0.55 | 0.9 | 0.8 | 1 | 0.9 | 1 | 0.9 | 0.9 | 1 | - | 0 |
|  | L | 0 | 0 | 0 | 0 | 0 | 0 | 0 | 0 | 0 | 0 | 0 | - |

| 3 - 7 million years ago | | | | | | | | | | | | | |
| --- | --- | --- | --- | --- | --- | --- | --- | --- | --- | --- | --- | --- | --- |
|  | | Destination | | | | | | | | | | | |
|  |  | A | B | C | D | E | F | G | H | I | J | K | L |
| From where they came | A | - | 0.75 | 0.15 | 0.15 | 0.1 | 0.1 | 0.1 | 0.1 | 0.1 | 0.1 | 0.1 | 0 |
|  | B | 0.75 | - | 1 | 1 | 0.8 | 0.55 | 0.55 | 0.35 | 0.35 | 0.45 | 0.65 | 0 |
|  | C | 0.15 | 0.75 | - | 0.9 | 0.9 | 0.65 | 0.65 | 0.45 | 0.45 | 0.55 | 0.65 | 0 |
|  | D | 0.15 | 0.75 | 0.9 | - | 0.9 | 0.65 | 0.75 | 0.45 | 0.45 | 0.45 | 0.65 | 0 |
|  | E | 0.1 | 0.55 | 0.9 | 0.9 | - | 0.85 | 0.85 | 0.65 | 0.65 | 0.65 | 0.85 | 0 |
|  | F | 0.1 | 0.45 | 0.8 | 0.8 | 1 | - | 1 | 1 | 0.9 | 0.9 | 0.9 | 0 |
|  | G | 0.1 | 0.45 | 0.8 | 0.8 | 1 | 1 | - | 1 | 1 | 1 | 1 | 0 |
|  | H | 0.1 | 0.1 | 0.2 | 0.2 | 0.4 | 0.75 | 0.75 | - | 1 | 0.65 | 0.55 | 0 |
|  | I | 0.1 | 0.1 | 0.2 | 0.2 | 0.4 | 0.65 | 0.75 | 1 | - | 1 | 0.75 | 0 |
|  | J | 0.1 | 0.1 | 0.3 | 0.2 | 0.4 | 0.65 | 0.75 | 0.65 | 1 | - | 0.85 | 0 |
|  | K | 0.1 | 0.65 | 1 | 0.8 | 1 | 0.9 | 1 | 0.9 | 0.9 | 1 | - | 0 |
|  | L | 0 | 0 | 0 | 0 | 0 | 0 | 0 | 0 | 0 | 0 | 0 | - |

| 7 - 10 million years ago | | | | | | | | | | | | | |  |
| --- | --- | --- | --- | --- | --- | --- | --- | --- | --- | --- | --- | --- | --- | --- |
|  | | Destination | | | | | | | | | | | |  |
|  |  | A | B | C | D | E | F | G | H | I | J | K | L | |
| From where they came | A | - | 0.35 | 0.1 | 0.15 | 0.1 | 0.1 | 0.1 | 0.1 | 0.1 | 0.1 | 0.1 | 0 | |
|  | B | 0.5 | - | 0.75 | 1 | 0.4 | 0.3 | 0.3 | 0.3 | 0.2 | 0.3 | 0.4 | 0 | |
|  | C | 0.15 | 0.85 | - | 0.5 | 1 | 0.9 | 0.9 | 0.8 | 0.8 | 0.9 | 1 | 0 | |
|  | D | 0.15 | 0.85 | 0.75 | - | 0.5 | 0.4 | 0.5 | 0.3 | 0.3 | 0.3 | 0.4 | 0 | |
|  | E | 0.1 | 0.25 | 0.75 | 0.5 | - | 1 | 1 | 0.9 | 0.9 | 0.9 | 1 | 0 | |
|  | F | 0.1 | 0.15 | 0.65 | 0.4 | 1 | - | 1 | 1 | 0.9 | 0.9 | 0.9 | 0 | |
|  | G | 0.1 | 0.15 | 0.65 | 0.4 | 1 | 1 | - | 1 | 1 | 1 | 1 | 0 | |
|  | H | 0.1 | 0.1 | 0.55 | 0.3 | 0.9 | 1 | 1 | - | 1 | 0.65 | 0.8 | 0 | |
|  | I | 0.1 | 0.1 | 0.55 | 0.3 | 0.9 | 0.9 | 1 | 1 | - | 1 | 0.9 | 0 | |
|  | J | 0.1 | 0.15 | 0.65 | 0.3 | 0.9 | 0.9 | 1 | 0.65 | 1 | - | 1 | 0 | |
|  | K | 0.1 | 0.25 | 0.75 | 0.4 | 1 | 0.9 | 1 | 0.9 | 0.9 | 1 | - | 0 | |
|  | L | 0 | 0 | 0 | 0 | 0 | 0 | 0 | 0 | 0 | 0 | 0 | - | |

| 10 - 23 million years ago | | | | | | | | | | | | | | |
| --- | --- | --- | --- | --- | --- | --- | --- | --- | --- | --- | --- | --- | --- | --- |
|  | | Destination | | | | | | | | | | | | |
|  |  | A | B | C | D | E | F | G | H | I | J | K | L |  |
| From where they came | A | - | 0.2 | 0.1 | 0.1 | 0.1 | 0.1 | 0.1 | 0.1 | 0.1 | 0.1 | 0 | 0 |  |
|  | B | 0.25 | - | 0.5 | 0.5 | 0.4 | 0.3 | 0.3 | 0.2 | 0.2 | 0.3 | 0.1 | 0 |  |
|  | C | 0.1 | 0.45 | - | 1 | 1 | 0.9 | 0.9 | 0.8 | 0.8 | 0.8 | 0.75 | 0 |  |
|  | D | 0.1 | 0.45 | 0.75 | - | 1 | 0.9 | 1 | 0.8 | 0.8 | 0.8 | 0.65 | 0 |  |
|  | E | 0.1 | 0.35 | 0.75 | 1 | - | 1 | 1 | 0.9 | 0.9 | 0.9 | 0.75 | 0 |  |
|  | F | 0.1 | 0.25 | 0.65 | 0.9 | 1 | - | 1 | 1 | 0.9 | 0.9 | 0.65 | 0 |  |
|  | G | 0.1 | 0.25 | 0.65 | 0.9 | 1 | 1 | - | 1 | 1 | 1 | 0.75 | 0 |  |
|  | H | 0.1 | 0.15 | 0.55 | 0.8 | 0.9 | 1 | 1 | - | 1 | 0.9 | 0.55 | 0 |  |
|  | I | 0.1 | 0.15 | 0.55 | 0.8 | 0.9 | 0.9 | 1 | 1 | - | 1 | 0.65 | 0 |  |
|  | J | 0.1 | 0.25 | 0.65 | 0.8 | 0.9 | 0.9 | 1 | 0.9 | 1 | - | 0.75 | 0 |  |
|  | K | 0.1 | 0.35 | 0.75 | 0.9 | 1 | 0.9 | 1 | 0.9 | 0.9 | 1 | - | 0 |  |
|  | L | 0 | 0 | 0 | 0 | 0 | 0 | 0 | 0 | 0 | 0 | 0 | - |  |

| 23 - 35 million years ago | | | | | | | | | | | | | | |
| --- | --- | --- | --- | --- | --- | --- | --- | --- | --- | --- | --- | --- | --- | --- |
|  | | Destination | | | | | | | | | | | | |
|  |  | A | B | C | D | E | F | G | H | I | J | K | L |  |
| From where they came | A | - | 0 | 0 | 0 | 0 | 0 | 0 | 0 | 0 | 0 | 0 | 0 |  |
|  | B | 0 | - | 1 | 1 | 0.9 | 0.8 | 0.8 | 0.7 | 0.7 | 0.8 | 0.9 | 0 |  |
|  | C | 0 | 1 | - | 1 | 1 | 0.9 | 0.9 | 0.8 | 0.8 | 0.9 | 1 | 0 |  |
|  | D | 0 | 1 | 1 | - | 1 | 0.9 | 1 | 0.8 | 0.8 | 0.8 | 0.9 | 0 |  |
|  | E | 0 | 0.9 | 1 | 1 | - | 1 | 1 | 0.9 | 0.9 | 0.9 | 1 | 0 |  |
|  | F | 0 | 0.8 | 0.9 | 0.9 | 1 | - | 1 | 1 | 0.9 | 0.9 | 0.9 | 0 |  |
|  | G | 0 | 0.8 | 0.9 | 0.9 | 1 | 1 | - | 1 | 1 | 1 | 1 | 0 |  |
|  | H | 0 | 0.7 | 0.8 | 0.8 | 0.9 | 1 | 1 | - | 1 | 0.9 | 0.8 | 0 |  |
|  | I | 0 | 0.7 | 0.8 | 0.8 | 0.9 | 0.9 | 1 | 1 | - | 1 | 0.9 | 0 |  |
|  | J | 0 | 0.8 | 0.9 | 0.8 | 0.9 | 0.9 | 1 | 0.9 | 1 | - | 1 | 0 |  |
|  | K | 0 | 0.9 | 1 | 0.9 | 1 | 0.9 | 1 | 0.9 | 0.9 | 1 | - | 0 |  |
|  | L | 0 | 0 | 0 | 0 | 0 | 0 | 0 | 0 | 0 | 0 | 0 | - |  |

| 35 - 45 million years ago | | | | | | | | | | | | | |  |
| --- | --- | --- | --- | --- | --- | --- | --- | --- | --- | --- | --- | --- | --- | --- |
|  | | Destination | | | | | | | | | | | |  |
|  |  | A | B | C | D | E | F | G | H | I | J | K | L | |
| From where they came | A | - | 0 | 0 | 0 | 0 | 0 | 0 | 0 | 0 | 0 | 0 | 0 | |
|  | B | 0 | - | 1 | 1 | 0.9 | 0.8 | 0.8 | 0.7 | 0.7 | 0.8 | 0.9 | 0 | |
|  | C | 0 | 1 | - | 1 | 1 | 0.9 | 0.9 | 0.8 | 0.8 | 0.9 | 1 | 0 | |
|  | D | 0 | 1 | 1 | - | 1 | 0.9 | 1 | 0.8 | 0.8 | 0.8 | 0.9 | 0 | |
|  | E | 0 | 0.9 | 1 | 1 | - | 1 | 1 | 0.9 | 0.9 | 0.9 | 1 | 0 | |
|  | F | 0 | 0.8 | 0.9 | 0.9 | 1 | - | 1 | 1 | 0.9 | 0.9 | 0.9 | 0 | |
|  | G | 0 | 0.8 | 0.9 | 0.9 | 1 | 1 | - | 1 | 1 | 1 | 1 | 0 | |
|  | H | 0 | 0.7 | 0.8 | 0.8 | 0.9 | 1 | 1 | - | 1 | 0.9 | 0.9 | 0 | |
|  | I | 0 | 0.7 | 0.8 | 0.8 | 0.9 | 0.9 | 1 | 1 | - | 1 | 0.9 | 0 | |
|  | J | 0 | 0.8 | 0.9 | 0.8 | 0.9 | 0.9 | 1 | 0.9 | 1 | - | 1 | 0 | |
|  | K | 0 | 0.9 | 1 | 0.9 | 1 | 0.9 | 1 | 0.9 | 0.9 | 1 | - | 0 | |
|  | L | 0 | 0 | 0 | 0 | 0 | 0 | 0 | 0 | 0 | 0 | 0 | - | |

| 45 - 55 million years ago | | | | | | | | | | | | | | |
| --- | --- | --- | --- | --- | --- | --- | --- | --- | --- | --- | --- | --- | --- | --- |
|  | | Destination | | | | | | | | | | | | |
|  |  | A | B | C | D | E | F | G | H | I | J | K | L |  |
| From where they came | A | - | 0 | 0 | 0 | 0 | 0 | 0 | 0 | 0 | 0 | 0 | 0 |  |
|  | B | 0 | - | 1 | 1 | 0.9 | 0.8 | 0.8 | 0.7 | 0.7 | 0.8 | 0.9 | 0.1 |  |
|  | C | 0 | 1 | - | 1 | 1 | 0.9 | 0.9 | 0.8 | 0.8 | 0.9 | 1 | 0.1 |  |
|  | D | 0 | 1 | 1 | - | 1 | 0.9 | 1 | 0.8 | 0.8 | 0.8 | 0.9 | 0.1 |  |
|  | E | 0 | 0.9 | 1 | 1 | - | 1 | 1 | 0.9 | 0.9 | 0.9 | 1 | 0.1 |  |
|  | F | 0 | 0.8 | 0.9 | 0.9 | 1 | - | 1 | 1 | 0.9 | 0.9 | 0.9 | 0.1 |  |
|  | G | 0 | 0.8 | 0.9 | 0.9 | 1 | 1 | - | 1 | 1 | 1 | 1 | 0.1 |  |
|  | H | 0 | 0.7 | 0.8 | 0.8 | 0.9 | 1 | 1 | - | 1 | 0.9 | 0.8 | 0.1 |  |
|  | I | 0 | 0.7 | 0.8 | 0.8 | 0.9 | 0.9 | 1 | 1 | - | 1 | 0.9 | 0.1 |  |
|  | J | 0 | 0.8 | 0.9 | 0.8 | 0.9 | 0.9 | 1 | 0.9 | 1 | - | 1 | 0.1 |  |
|  | K | 0 | 0.9 | 1 | 0.9 | 1 | 0.9 | 1 | 0.9 | 0.9 | 1 | - | 0.2 |  |
|  | L | 0 | 0.1 | 0.1 | 0.1 | 0.1 | 0.1 | 0.1 | 0.1 | 0.1 | 0.1 | 0.2 | - |  |

**REFERENCES**

**Bacon CD, Mora A, Wagner WL, Jaramillo CA**. **2013**. Testing geological models of evolution of the Isthmus of Panama in a phylogenetic framework. *Botanical Journal of the Linnean Society* 171: 287–300.

**Bacon CD, Silvestro D, Jaramillo CA, Smith BT, Chakrabarty P, Antonelli A**. **2015**. Biological evidence supports an early and complex emergence of the Isthmus of Panama. *Proceedings of the National Academy of Sciences* 112: 6110–6115.

**Boschman LM, Condamine FL**. **2022**. Mountain radiations are not only rapid and recent: Ancient diversification of South American frog and lizard families related to Paleogene Andean orogeny and Cenozoic climate variations. *Global and Planetary Change* 208: 103704.

**Farris DW, Jaramillo CA, Bayona G, Restrepo-Moreno SA, Montes C, Cardona A, Mora A, Speakman RJ, Glascock MD, Valencia V**. **2011**. Fracturing of the Panamanian Isthmus during initial collision with: South America. *Geology* 39: 1007–1010.

**Gregory-Wodzicki KM**. **2000**. Uplift history of the Central and Northern Andes: A review. *Geological Society of America Bulletin* 112: 1091–1105.

**Hernández RM, Jordan TE, Dalenz Farjat A, Echavarría L, Idleman BD, Reynolds JH, Farjat AD, Echavarría L, Idleman BD, Reynolds JH**. **2005**. Age, distribution, tectonics, and eustatic controls of the Paranense and Caribbean marine transgressions in southern Bolivia and Argentina. *Journal of South American Earth Sciences* 19: 495–512.

**Hoorn C**. **1993**. Marine incursions and the influence of Andean tectonics on the Miocene depositional history of northwestern Amazonia: results of a palynostratigraphic study. *Palaeogeography, Palaeoclimatology, Palaeoecology* 105: 267–309.

**Hoorn C, Wesselingh FP, ter Steege H, Bermudez MA, Mora A, Sevink J, Sanmartín I, Sanchez-Meseguer A, Anderson CL, Figueiredo JP, Jaramillo CA, Riff D, Negri FR, Hooghiemstra H, Lundberg JG, Stadler T, Sarkinen T, Antonelli A**. **2010**. Amazonia Through Time: Andean Uplift, Climate Change, Landscape Evolution, and Biodiversity. *Science* 330: 927–931.

**Jaramillo CA, Montes C, Cardona A, Silvestro D, Antonelli A, Bacon CD**. **2017**. Comment (1) on “Formation of the Isthmus of Panama” by O’Dea et al. *Science Advances* 3: 1–8.

**Matzke NJ**. **2013**. Probabilistic historical biogeography:new models for founder-event speciation, eimperfect detection, and fossils allow improved accurancy and model-testing. *Frontiers of Biogeography* 5: 242–248.

**Matzke NJ**. **2014**. Model selection in historical biogeography reveals that founder-event speciation is a crucial process in island clades. *Systematic Biology* 63: 951–970.

**Salas-Gismondi R, Flynn JJ, Baby P, Wesselingh FP, Antoine PO, Salas-gismondi R**. **2015**. A Miocene hyperdiverse crocodylian community reveals peculiar trophic dynamics in proto-Amazonian mega-wetlands. *Proceedings of the Royal Society B: Biological Sciences* 282: 4–8.

**Scotese CR**. **2021**. An atlas of Phanerozoic paleogeographic maps: the seas come in and the seas go out. *Annual Review of Earth and Planetary Sciences* 49: 679–728.

**Souza DH, Hackspacher PC, Silva B V, Siqueira-Ribeiro MC, Hiruma ST**. **2021**. Temporal and spatial denudation trends in the continental margin of southeastern Brazil. *Journal of South American Earth Sciences* 105: 102931.
