# Supplementary figures and images for: Marine introgressions and Andean uplift have driven diversification in neotropical Monkey tree frogs (Anura, Phyllomedusinae)
Source: PeerJ. 2024 Apr 16;12:e17232. doi: 10.7717/peerj.17232 (PMC11027904; doi:10.7717/peerj.17232)

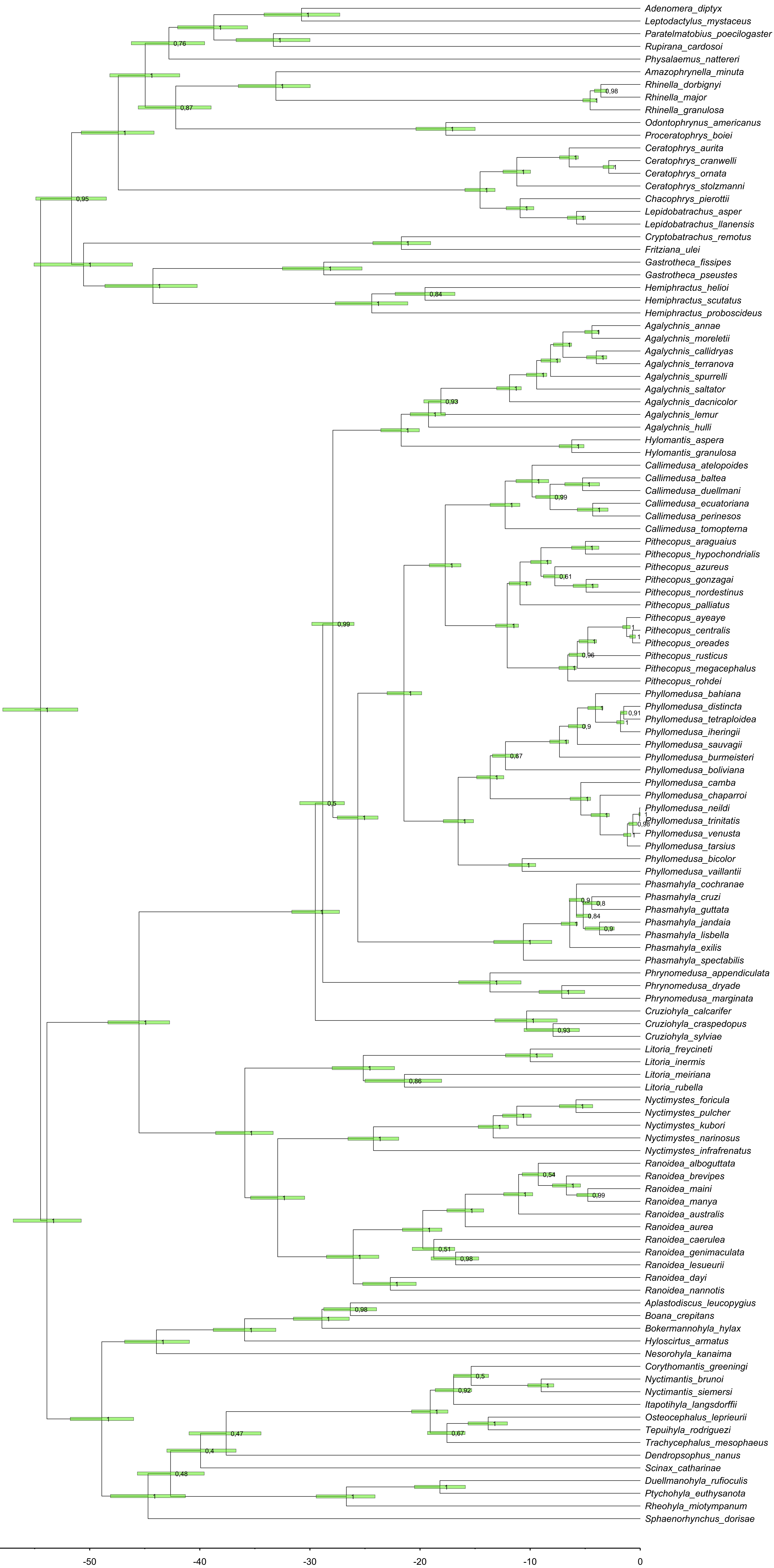

Supplement: Supplemental Information 5 — Horizontal green bars represent the 95% HPD (height posterior density) intervals for the divergence date estimates. Numbers in nodes indicate detailed posterior probabilities. [file peerj-12-17232-s005.pdf]
