# Supplementary material for: Marine introgressions and Andean uplift have driven diversification in neotropical Monkey tree frogs (Anura, Phyllomedusinae)
Source: PeerJ. 2024 Apr 16;12:e17232. doi: 10.7717/peerj.17232 (PMC11027904; doi:10.7717/peerj.17232)
Supplement: Supplemental Information 6 — Boxes with letters indicate the actual range of distribution of the species. Pie charts at nodes and descendants shows all the range probabilities reconstructed by the model. Colors also represent units/combination of units (see Fig. 1 for more details). Vertical dashed lines represent the timeslices from the time-stratified dispersal matrix. Observe that highly ambiguous nodes (such as the first one) became black. [file peerj-12-17232-s006.pdf]

Legend

_ A B C D E F G H I J K L AB AC AD AE AF AG AH AI AJ AK AL BC BD BE BF BG BH BI BJ BK BL CD CE CF CG CH CI CJ CK CL DE DF DG DH DI DJ DK DL EF EG EH EI EJ EK EL FG FH FI FJ FK FL GH GI GJ GK GL HI HJ HK HL

IJ IK IL JK JL KL ABC ABD ABE ABF ABG ABH ABI ABJ ABK ABL ACD ACE ACF ACG ACH ACI ACJ ACK ACL ADE ADF ADG ADH ADI ADJ ADK ADL AEF AEG AEH AEI AEJ AEK AEL AFG AFH AFI AFJ AFK AFL AGH AGI AGJ AGK AGL AHI AHJ AHK AHL AIJ AIK AIL AJK AJL AKL BCD BCE BCF BCG BCH BCI BCJ BCK BCL BDE BDF BDG

BDH BDI BDJ BDK BDL BEF BEG BEH BEI BEJ BEK BEL BFG BFH BFI BFJ BFK BFL BGH BGI BGJ BGK BGL BHI BHJ BHK BHL BIJ BIK BIL BJK BJL BKL CDE CDF CDG CDH CDI CDJ CDK CDL CEF CEG CEH CEI CEJ CEK CEL CFG CFH CFI CFJ CFK CFL CGH CGI CGJ CGK CGL CHI CHJ CHK CHL CIJ CIK CIL CJK CJL CKL DEF DEG DEH DEI

DEJ DEK DEL DFG DFH DFI DFJ DFK DFL DGH DGI DGJ DGK DGL DHI DHJ DHK DHL DIJ DIK DIL DJK DJL DKL EFG EFH EFI EFJ EFK EFL EGH EGI EGJ EGK EGL EHI EHJ EHK EHL EIJ EIK EIL EJK EJL EKL FGH FGI FGJ FGK FGL FHI FHJ FHK FHL FIJ FIK FIL FJK FJL FKL GHI GHJ GHK GHL GIJ GIK GIL GJK GJL GKL HIJ HIK HIL

HJK HJL HKL IJK IJL IKL JKL ABCD ABCE ABCF ABCG ABCH ABCI ABCJ ABCK ABCL ABDE ABDF ABDG ABDH ABDI ABDJ ABDK ABDL ABEF ABEG ABEH ABEI ABEJ ABEK ABEL ABFG ABFH ABFI ABFJ ABFK ABFL ABGH ABGI ABGJ ABGK ABGL ABHI ABHJ ABHK ABHL ABIJ ABIK ABIL ABJK ABJL ABKL ACDE ACDF ACDG ACDH ACDI ACDJ ACDK ACDL ACEF ACEG ACEH ACEI ACEJ ACEK ACEL ACFG ACFH ACFI ACFJ ACFK ACFL

ACGH ACGI ACGJ ACGK ACGL ACHI ACHJ ACHK ACHL ACIJ ACIK ACIL ACJK ACJL ACKL ADEF ADEG ADEH ADEI ADEJ ADEK ADEL ADFG ADFH ADFI ADFJ ADFK ADFL ADGH ADGI ADGJ ADGK ADGL ADHI ADHJ ADHK ADHL ADIJ ADIK ADIL ADJK ADJL ADKL AEFG AEFH AEFI AEFJ AEFK AEFL AEGH AEGI AEGJ AEGK AEGL AEHI AEHJ AEHK AEHL AEIJ AEIK AEIL AEJK AEJL AEKL AFGH AFGI AFGJ AFGK AFGL AFHI AFHJ AFHK AFHL

AFIJ AFIK AFIL AFJK AFJL AFKL AGHI AGHJ AGHK AGHL AGIJ AGIK AGIL AGJK AGJL AGKL AHIJ AHIK AHIL AHJK AHJL AHKL AIJK AIJL AIKL AJKL BCDE BCDF BCDG BCDH BCDI BCDJ BCDK BCDL BCEF BCEG BCEH BCEI BCEJ BCEK BCEL BCFG BCFH BCFI BCFJ BCFK BCFL BCGH BCGI BCGJ BCGK BCGL BCHI BCHJ BCHK BCHL BCIJ BCIK BCIL BCJK BCJL BCKL BDEF BDEG BDEH BDEI BDEJ BDEK BDEL BDFG BDFH BDFI BDFJ

BDFK BDFL BDGH BDGI BDGJ BDGK BDGL BDHI BDHJ BDHK BDHL BDIJ BDIK BDIL BDJK BDJL BDKL BEFG BEFH BEFI BEFJ BEFK BEFL BEGH BEGI BEGJ BEGK BEGL BEHI BEHJ BEHK BEHL BEIJ BEIK BEIL BEJK BEJL BEKL BFGH BFGI BFGJ BFGK BFGL BFHI BFHJ BFHK BFHL BFIJ BFIK BFIL BFJK BFJL BFKL BGHI BGHJ BGHK BGHL BGIJ BGIK BGIL BGJK BGJL BGKL BHIJ BHIK BHIL BHJK BHJL BHKL BIJK BIJL BIKL BJKL

CDEF CDEG CDEH CDEI CDEJ CDEK CDEL CDFG CDFH CDFI CDFJ CDFK CDFL CDGH CDGI CDGJ CDGK CDGL CDHI CDHJ CDHK CDHL CDIJ CDIK CDIL CDJK CDJL CDKL CEFG CEFH CEFI CEFJ CEFK CEFL CEGH CEGI CEGJ CEGK CEGL CEHI CEHJ CEHK CEHL CEIJ CEIK CEIL CEJK CEJL CEKL CFGH CFGI CFGJ CFGK CFGL CFHI CFHJ CFHK CFHL CFIJ CFIK CFIL CFJK CFJL CFKL CGHI CGHJ CGHK CGHL CGIJ CGIK CGIL CGJK CGJL

CGKL CHIJ CHIK CHIL CHJK CHJL CHKL CIJK CIJL CIKL CJKL DEFG DEFH DEFI DEFJ DEFK DEFL DEGH DEGI DEGJ DEGK DEGL DEHI DEHJ DEHK DEHL DEIJ DEIK DEIL DEJK DEJL DEKL DFGH DFGI DFGJ DFGK DFGL DFHI DFHJ DFHK DFHL DFIJ DFIK DFIL DFJK DFJL DFKL DGHI DGHJ DGHK DGHL DGIJ DGIK DGIL DGJK DGJL DGKL DHIJ DHIK DHIL DHJK DHJL DHKL DIJK DIJL DIKL DJKL EFGH EFGI EFGJ EFGK EFGL EFHI

EFHJ EFHK EFHL EFIJ EFIK EFIL EFJK EFJL EFKL EGHI EGHJ EGHK EGHL EGIJ EGIK EGIL EGJK EGJL EGKL EHIJ EHIK EHIL EHJK EHJL EHKL EIJK EIJL EIKL EJKL FGHI FGHJ FGHK FGHL FGIJ FGIK FGIL FGJK FGJL FGKL FHIJ FHIK FHIL FHJK FHJL FHKL FIJK FIJL FIKL FJKL GHIJ GHIK GHIL GHJK GHJL GHKL GIJK GIJL GIKL GJKL HIJK HIJL HIKL HJKL IJKL

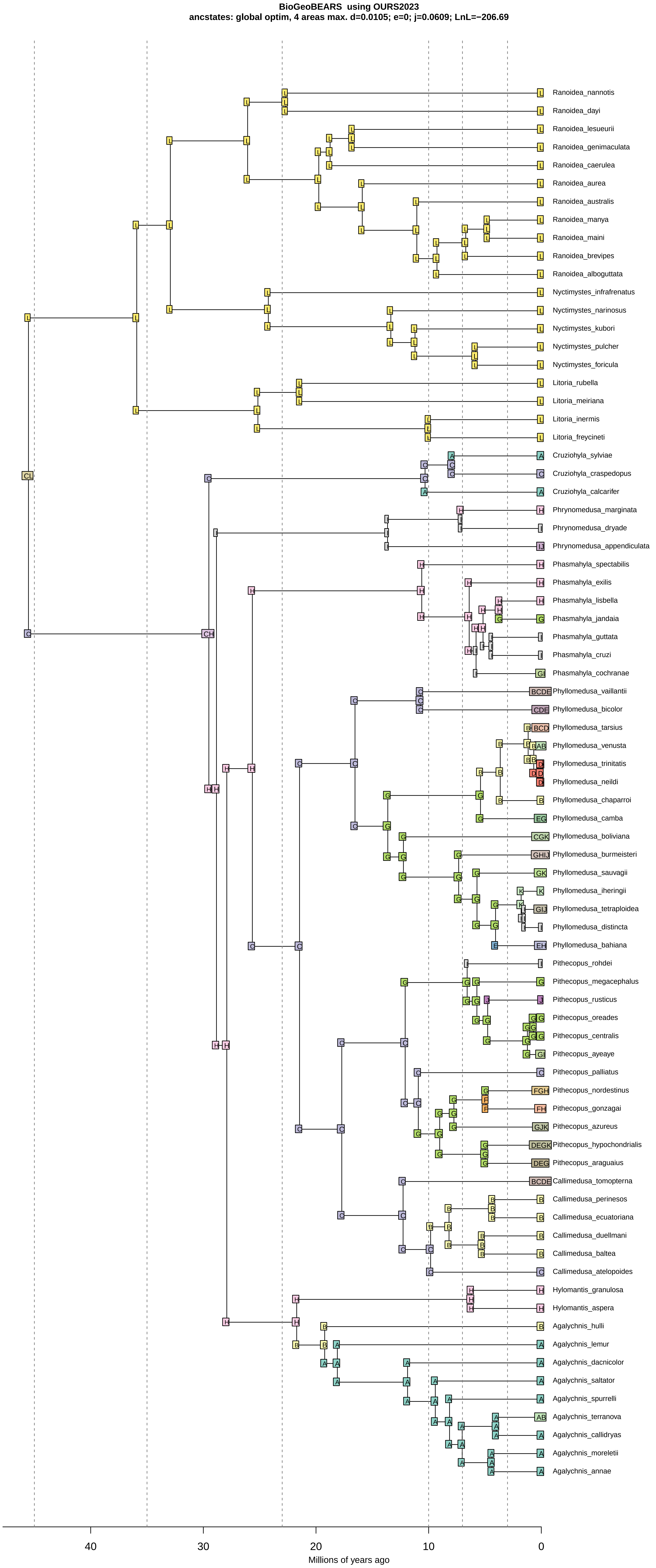
BioGeoBEARS using OURS2023
ancstates: global optim, 4 areas max. d=0.0105; e=0; j=0.0609; LnL=−206.69
Ranoidea_nannotis
Ranoidea_dayi
Ranoidea_lesueurii
Ranoidea_genimaculata
Ranoidea_caerulea
Ranoidea_aurea
Ranoidea_australis
Ranoidea_manya
Ranoidea_maini
Ranoidea_brevipes
Ranoidea_alboguttata
Nyctimystes_infrafrenatus
Nyctimystes_narinosus
Nyctimystes_kubori
Nyctimystes_pulcher
Nyctimystes_foricula
Litoria_rubella
Litoria_meiriana
Litoria_inermis
Litoria_freycineti
Cruziohyla_sylviae
Cruziohyla_craspedopus
Cruziohyla_calcarifer
Phrynomedusa_marginata
Phrynomedusa_dryade
Phrynomedusa_appendiculata
Phasmahyla_spectabilis
Phasmahyla_exilis
Phasmahyla_lisbella
Phasmahyla_jandaia
Phasmahyla_guttata
Phasmahyla_cruzi
Phasmahyla_cochranae
Phyllomedusa_vaillantii
Phyllomedusa_bicolor
Phyllomedusa_tarsius
Phyllomedusa_venusta
Phyllomedusa_trinitatis
Phyllomedusa_neildi
Phyllomedusa_chaparroi
Phyllomedusa_camba
Phyllomedusa_boliviana
Phyllomedusa_burmeisteri
Phyllomedusa_sauvagii
Phyllomedusa_iheringii
Phyllomedusa_tetraploidea
Phyllomedusa_distincta
Phyllomedusa_bahiana
Pithecopus_rohdei
Pithecopus_megacephalus
Pithecopus_rusticus
Pithecopus_oreades
Pithecopus_centralis
Pithecopus_ayeaye
Pithecopus_palliatus
Pithecopus_nordestinus
Pithecopus_gonzagai
Pithecopus_azureus
Pithecopus_hypochondrialis
Pithecopus_araguaius
Callimedusa_tomopterna
Callimedusa_perinesos
Callimedusa_ecuatoriana
Callimedusa_duellmani
Callimedusa_baltea
Callimedusa_atelopoides
Hylomantis_granulosa
Hylomantis_aspera
Agalychnis_hulli
Agalychnis_lemur
Agalychnis_dacnicolor
Agalychnis_saltator
Agalychnis_spurrelli
Agalychnis_terranova
Agalychnis_callidryas
Agalychnis_moreletii
Agalychnis_annae
40
30
20
10
0
Millions of years ago

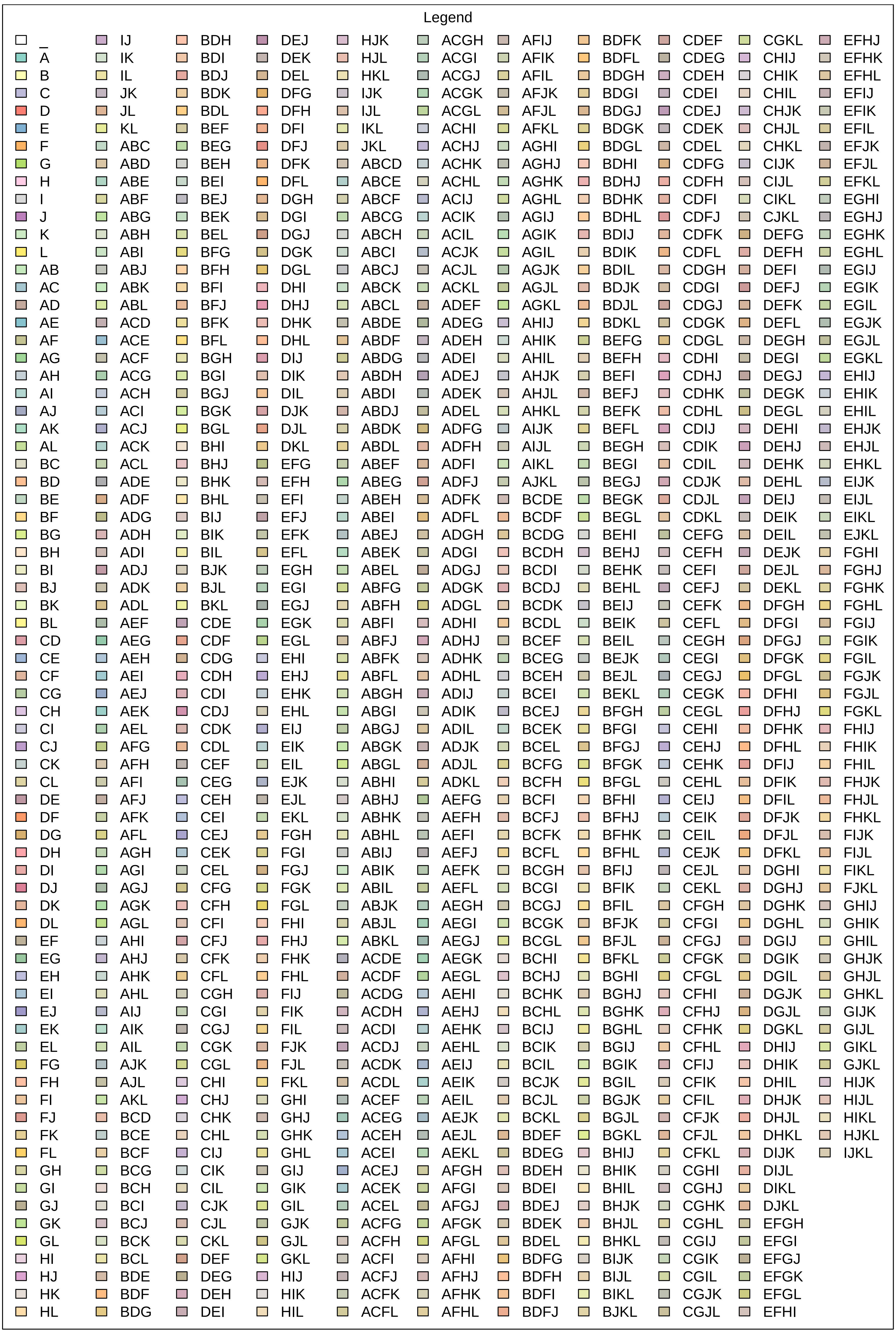
Legend
_ A B C D E F G H I J K L AB AC AD AE AF AG AH AI AJ AK AL BC BD BE BF BG BH BI BJ BK BL CD CE CF CG CH CI CJ CK CL DE DF DG DH DI DJ DK DL EF EG EH EI EJ EK EL FG FH FI FJ FK FL GH GI GJ GK GL HI HJ HK HL
IJ IK IL JK JL KL ABC ABD ABE ABF ABG ABH ABI ABJ ABK ABL ACD ACE ACF ACG ACH ACI ACJ ACK ACL ADE ADF ADG ADH ADI ADJ ADK ADL AEF AEG AEH AEI AEJ AEK AEL AFG AFH AFI AFJ AFK AFL AGH AGI AGJ AGK AGL AHI AHJ AHK AHL AIJ AIK AIL AJK AJL AKL BCD BCE BCF BCG BCH BCI BCJ BCK BCL BDE BDF BDG
BDH BDI BDJ BDK BDL BEF BEG BEH BEI BEJ BEK BEL BFG BFH BFI BFJ BFK BFL BGH BGI BGJ BGK BGL BHI BHJ BHK BHL BIJ BIK BIL BJK BJL BKL CDE CDF CDG CDH CDI CDJ CDK CDL CEF CEG CEH CEI CEJ CEK CEL CFG CFH CFI CFJ CFK CFL CGH CGI CGJ CGK CGL CHI CHJ CHK CHL CIJ CIK CIL CJK CJL CKL DEF DEG DEH DEI
DEJ DEK DEL DFG DFH DFI DFJ DFK DFL DGH DGI DGJ DGK DGL DHI DHJ DHK DHL DIJ DIK DIL DJK DJL DKL EFG EFH EFI EFJ EFK EFL EGH EGI EGJ EGK EGL EHI EHJ EHK EHL EIJ EIK EIL EJK EJL EKL FGH FGI FGJ FGK FGL FHI FHJ FHK FHL FIJ FIK FIL FJK FJL FKL GHI GHJ GHK GHL GIJ GIK GIL GJK GJL GKL HIJ HIK HIL
HJK HJL HKL IJK IJL IKL JKL ABCD ABCE ABCF ABCG ABCH ABCI ABCJ ABCK ABCL ABDE ABDF ABDG ABDH ABDI ABDJ ABDK ABDL ABEF ABEG ABEH ABEI ABEJ ABEK ABEL ABFG ABFH ABFI ABFJ ABFK ABFL ABGH ABGI ABGJ ABGK ABGL ABHI ABHJ ABHK ABHL ABIJ ABIK ABIL ABJK ABJL ABKL ACDE ACDF ACDG ACDH ACDI ACDJ ACDK ACDL ACEF ACEG ACEH ACEI ACEJ ACEK ACEL ACFG ACFH ACFI ACFJ ACFK ACFL
ACGH ACGI ACGJ ACGK ACGL ACHI ACHJ ACHK ACHL ACIJ ACIK ACIL ACJK ACJL ACKL ADEF ADEG ADEH ADEI ADEJ ADEK ADEL ADFG ADFH ADFI ADFJ ADFK ADFL ADGH ADGI ADGJ ADGK ADGL ADHI ADHJ ADHK ADHL ADIJ ADIK ADIL ADJK ADJL ADKL AEFG AEFH AEFI AEFJ AEFK AEFL AEGH AEGI AEGJ AEGK AEGL AEHI AEHJ AEHK AEHL AEIJ AEIK AEIL AEJK AEJL AEKL AFGH AFGI AFGJ AFGK AFGL AFHI AFHJ AFHK AFHL
AFIJ AFIK AFIL AFJK AFJL AFKL AGHI AGHJ AGHK AGHL AGIJ AGIK AGIL AGJK AGJL AGKL AHIJ AHIK AHIL AHJK AHJL AHKL AIJK AIJL AIKL AJKL BCDE BCDF BCDG BCDH BCDI BCDJ BCDK BCDL BCEF BCEG BCEH BCEI BCEJ BCEK BCEL BCFG BCFH BCFI BCFJ BCFK BCFL BCGH BCGI BCGJ BCGK BCGL BCHI BCHJ BCHK BCHL BCIJ BCIK BCIL BCJK BCJL BCKL BDEF BDEG BDEH BDEI BDEJ BDEK BDEL BDFG BDFH BDFI BDFJ
BDFK BDFL BDGH BDGI BDGJ BDGK BDGL BDHI BDHJ BDHK BDHL BDIJ BDIK BDIL BDJK BDJL BDKL BEFG BEFH BEFI BEFJ BEFK BEFL BEGH BEGI BEGJ BEGK BEGL BEHI BEHJ BEHK BEHL BEIJ BEIK BEIL BEJK BEJL BEKL BFGH BFGI BFGJ BFGK BFGL BFHI BFHJ BFHK BFHL BFIJ BFIK BFIL BFJK BFJL BFKL BGHI BGHJ BGHK BGHL BGIJ BGIK BGIL BGJK BGJL BGKL BHIJ BHIK BHIL BHJK BHJL BHKL BIJK BIJL BIKL BJKL
CDEF CDEG CDEH CDEI CDEJ CDEK CDEL CDFG CDFH CDFI CDFJ CDFK CDFL CDGH CDGI CDGJ CDGK CDGL CDHI CDHJ CDHK CDHL CDIJ CDIK CDIL CDJK CDJL CDKL CEFG CEFH CEFI CEFJ CEFK CEFL CEGH CEGI CEGJ CEGK CEGL CEHI CEHJ CEHK CEHL CEIJ CEIK CEIL CEJK CEJL CEKL CFGH CFGI CFGJ CFGK CFGL CFHI CFHJ CFHK CFHL CFIJ CFIK CFIL CFJK CFJL CFKL CGHI CGHJ CGHK CGHL CGIJ CGIK CGIL CGJK CGJL
CGKL CHIJ CHIK CHIL CHJK CHJL CHKL CIJK CIJL CIKL CJKL DEFG DEFH DEFI DEFJ DEFK DEFL DEGH DEGI DEGJ DEGK DEGL DEHI DEHJ DEHK DEHL DEIJ DEIK DEIL DEJK DEJL DEKL DFGH DFGI DFGJ DFGK DFGL DFHI DFHJ DFHK DFHL DFIJ DFIK DFIL DFJK DFJL DGHI DGHJ DGHK DGHL DGIJ DGIK DGIL DGJK DGJL DGKL DHIJ DHIK DHIL DHJK DHJL DHKL DIJK DIJL DIKL DJKL EFGH EFGI EFGJ EFGK EFGL EFHI
EFHJ EFHK EFHL EFIJ EFIK EFIL EFJK EFJL EFKL EGHI EGHJ EGHK EGHL EGIJ EGIK EGIL EGJK EGJL EGKL EHIJ EHIK EHIL EHJK EHJL EHKL EIJK EIJL EIKL EJKL FGHI FGHJ FGHK FGHL FGIJ FGIK FGIL FGJK FGJL FGKL FHIJ FHIK FHIL FHJK FHJL FHKL FIJK FIJL FIKL FJKL GHIJ GHIK GHIL GHJK GHJL GHKL GIJK GIJL GIKL GJKL HIJK HIJL HIKL HJKL IJKL

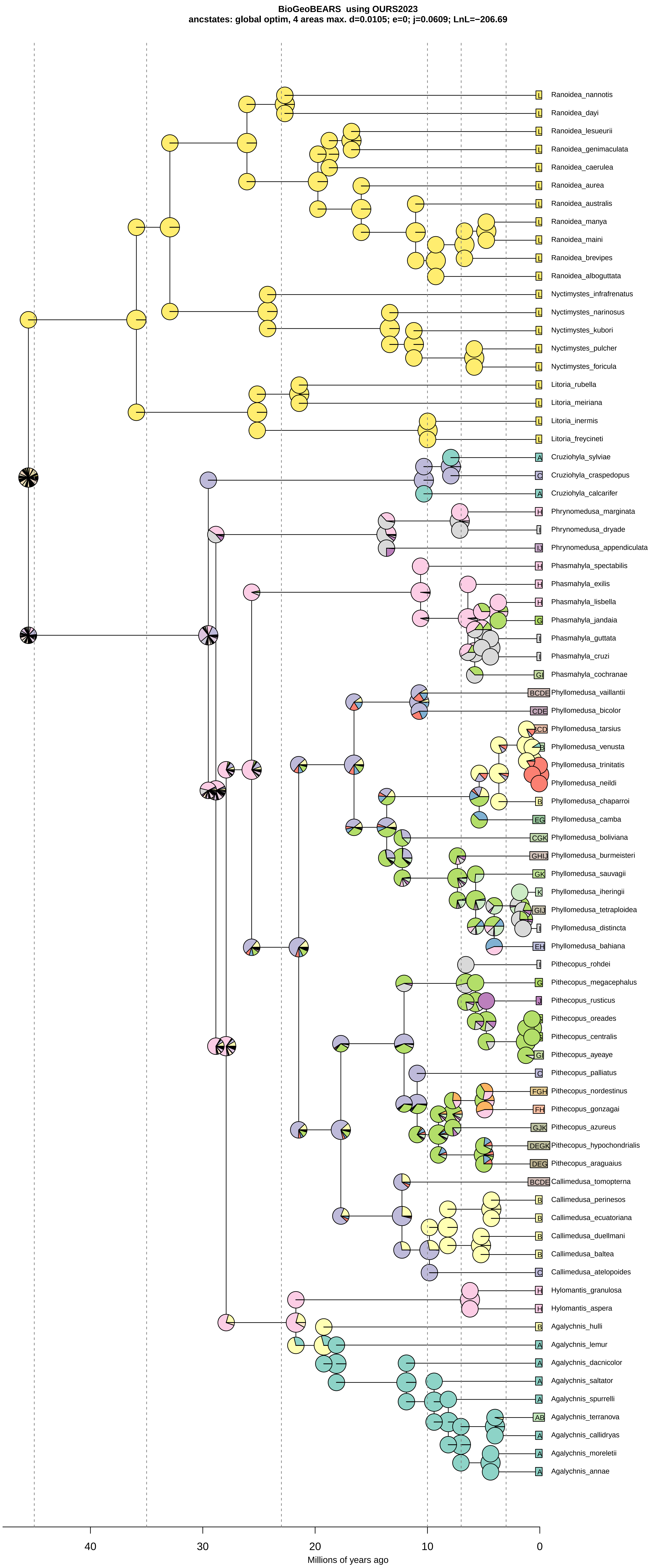
BioGeoBEARS using OURS2023
ancstates: global optim, 4 areas max. d=0.0105; e=0; j=0.0609; LnL=−206.69
Ranoidea_nannotis
Ranoidea_dayi
Ranoidea_lesueurii
Ranoidea_genimaculata
Ranoidea_caerulea
Ranoidea_aurea
Ranoidea_australis
Ranoidea_manya
Ranoidea_maini
Ranoidea_brevipes
Ranoidea_alboguttata
Nyctimystes_infrafrenatus
Nyctimystes_narinosus
Nyctimystes_kubori
Nyctimystes_pulcher
Nyctimystes_foricula
Litoria_rubella
Litoria_meiriana
Litoria_inermis
Litoria_freycineti
Cruziohyla_sylviae
Cruziohyla_craspedopus
Cruziohyla_calcarifer
Phrynomedusa_marginata
Phrynomedusa_dryade
Phrynomedusa_appendiculata
Phasmahyla_spectabilis
Phasmahyla_exilis
Phasmahyla_lisbella
Phasmahyla_jandaia
Phasmahyla_guttata
Phasmahyla_cruzi
Phasmahyla_cochranae
Phyllomedusa_vaillantii
Phyllomedusa_bicolor
Phyllomedusa_tarsius
Phyllomedusa_venusta
Phyllomedusa_trinitatis
Phyllomedusa_neildi
Phyllomedusa_chaparroi
Phyllomedusa_camba
Phyllomedusa_boliviana
Phyllomedusa_burmeisteri
Phyllomedusa_sauvagii
Phyllomedusa_iheringii
Phyllomedusa_tetraploidea
Phyllomedusa_distincta
Phyllomedusa_bahiana
Pithecopus_rohdei
Pithecopus_megacephalus
Pithecopus_rusticus
Pithecopus_oreades
Pithecopus_centralis
Pithecopus_ayeaye
Pithecopus_palliatus
Pithecopus_nordestinus
Pithecopus_gonzagai
Pithecopus_azureus
Pithecopus_hypochondrialis
Pithecopus_araguaius
Callimedusa_tomopterna
Callimedusa_perinesos
Callimedusa_ecuatoriana
Callimedusa_duellmani
Callimedusa_baltea
Callimedusa_atelopoides
Hylomantis_granulosa
Hylomantis_aspera
Agalychnis_hulli
Agalychnis_lemur
Agalychnis_dacnicolor
Agalychnis_saltator
Agalychnis_spurrelli
Agalychnis_terranova
Agalychnis_callidryas
Agalychnis_moreletii
Agalychnis_annae
40
30
20
10
0
Millions of years ago
